# Tailoring full-Stokes thermal emission from twisted-gratings structures

**DOI:** 10.1515/nanoph-2023-0395

**Published:** 2023-10-25

**Authors:** Chiyu Yang, Wenshan Cai, Zhuomin M. Zhang

**Affiliations:** George W. Woodruff School of Mechanical Engineering Georgia Institute of Technology, Atlanta, GA 30332, USA; School of Electrical and Computer Engineering, Georgia Institute of Technology, Atlanta, GA 30332, USA

**Keywords:** circular polarization, full-Stokes thermal emission, metamaterial, twisted gratings

## Abstract

Polarized thermal emission finds extensive applications in remote sensing, landmine detection, and target detection. In applications such as ellipsometry and biomedical analysis, the generation of emission with controllable polarization is preferred. It is desired to manipulate the polarization state over the full Stokes parameters. While numerous studies have demonstrated either linear or circular polarization control using metamaterials, full-Stokes thermal emission has not been explored. Here, a microstructure based on two layers of silicon carbide gratings is proposed to tailor the polarization state of thermal emission, covering the full-Stokes parameter range. The bilayer twisted-gratings structure breaks mirror symmetry. Wave interference at the interfaces and diffraction by the gratings enhance the emission dichroism, resulting in almost completely polarized emission. By adjusting the twist angle between the gratings, the polarization state can be continuously tuned from linear to circular, nearly covering the entire surface of Poincaré sphere. This study provides a design for tailoring full-Stokes emission with notable advantages over other plasmonic metasurfaces.

## 1 Introduction

Thermal emission is often considered to be incoherent, broadband, and unpolarized [1]. Unlike visible and near-infrared sources, which are predominantly achieved using semiconductors, mid-infrared emission is typically generated thermally dictated by Planck’s law. Over the past decades, metasurfaces made of subwavelength micro/nanostructured materials have attracted significant attention due to their unique and tunable thermal emission properties [2–7]. This opens up possibilities for controlling polarized thermal emission in compact and miniaturized devices. For instance, linearly polarized emission can be produced using relatively simple grating structures [5], while circularly polarized emission is typically generated through symmetry-breaking structures [8, 9] or nonreciprocal materials such as magneto-optical materials [10], Weyl semimetals [11, 12], and others.

Current studies on polarized thermal emitters are primarily focused on either linear or circular polarization. Generating arbitrary elliptical polarization requires the full control of thermal emission across the Poincaré sphere. In recent years, metasurfaces have been employed to realize full-Stokes polarization of visible and infrared lights in transmission [13–15], reflection [16], and absorption modes [17–19], providing unprecedented opportunities in the applications of imaging, holograms, quantum information processing, and optical communications. Despite these advancements, polarized thermal emission with arbitrary ellipticity in the midinfrared region has remained largely unexplored. Polarized thermal emission sources may have applications in drug analysis [20], midinfrared imaging [21, 22], and ellipsometry [23]. Nevertheless, the tunability of most metasurfaces for full-Stokes polarization is limited due to their geometrical complexity. For instance, ellipsometry requires four independent polarization states for incidence, and this limitation may hinder the utilization of metasurfaces in related applications.

**Figure 1:**
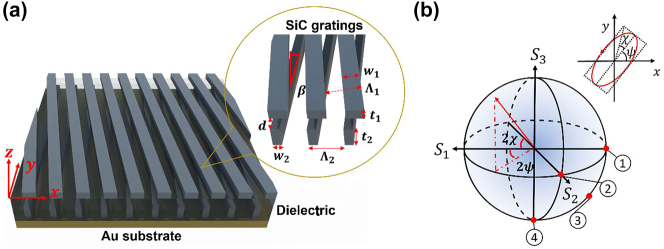
Illustration of the emitter and the Poincaré sphere. (a) The proposed emitter is composed of two gratings. The top SiC grating whose slits are filled with air is placed on a dielectric film (*n* = 1.5). The bottom SiC grating whose slits are filled with the same dielectric is placed on an opaque gold substrate. The inset shows the enlarged gratings. The following geometrical parameters are used in the calculations: Λ_1_ = Λ_2_ = 1 µm, *w*_1_ = 0.54 µm, *w*_2_ = 0.39 µm, *t*_1_ = 0.60 µm, *t*_2_ = 0.96 µm, and *d* = 0.58 µm, while the twist angle *β* is allowed to vary between 0° and 11.7°. (b) The polarization ellipse (upper-right) and the Poincaré sphere with rotation angle *ψ* (0 ≤ *ψ* < *π*) and the ellipticity *χ* (–*π*/4 ≤ *χ* < *π*/4) are depicted. The polarization of thermal emission can be tailored over the Poincaré sphere (as indicated by states 1 and 2 [linear], 3 [elliptical], and 4 [circular]).

Here, a full-Stokes thermal emitter with desired polarization states in the mid-infrared region is proposed. The design is based on two silicon carbide gratings with different strip widths, stacked on a metal substrate, as illustrated in Figure 1a. A twist angle between the top and the bottom grating is introduced to break mirror symmetry. The twisted-gratings structure exhibits a high degree of polarization in thermal emission. Adjusting the twist angle allows for changing the ellipticity of the polarized emission while keeping the spectral emission peak wavelength unaltered. By varying the twist angle, the polarization state for normal emission can be continuously tuned from linear to circular, nearly covering the entire surface of Poincaré sphere, as shown in Figure 1b. Although multilayer gratings structures [24] or multilayer antenna structures [25, 26] have been proposed to achieve circularly polarized light control, the potential of utilizing a twisted-gratings structure as a full-Stokes emitter has not been explored. This study presents a simple design to actualize the full-Stokes thermal emission with desired polarization states with prominent advantages over other plasmonic metasurfaces. Moreover, it holds promise for integration into micro-electromechanical systems (MEMS) to actively control thermal emission.

## 2 Results and discussion

Twisted bilayer structures have recently been proposed as a means of tuning the optical resonance frequency [27, 28]. The advancement in dynamic optical metamaterials has demonstrated the feasibility of modifying the geometrical parameters through mechanical actuations, such as tunable piezoelectric-based waveplates [16] and lens [29]. Here, gratings structure is used as the building block, and silicon carbide (SiC) is selected as the material for the gratings due to its low thermal expansion and superior heat resistance. The grating period and grating width can be precisely controlled using interference lithography by adjusting the angle between the interfering beams and the laser intensity [30], thus enabling the fabrication of the design with a significant level of precision in its dimensions. The proposed emitter shown in Figure 1a consists of two gratings with the same lattice constant or period, Λ_1_ = Λ_2_ = 1 µm. The gratings are made of SiC with strip widths of *w*_1_ = 0.54 µm and *w*_2_ = 0.39 µm, respectively. The top grating of thickness *t*_1_ = 0.60 µm is placed on a dielectric film whose thickness is *d* = 0.58 µm, and the grating of thickness *t*_2_ = 0.96 µm below the substrate is placed on a thick (opaque) gold substrate. Gold is used as the ground plane due to its high reflectance and opacity, which gives a very low emissivity in the region where the double-layer gratings are transparent. Furthermore, the gold layer enhances the emissivity peak due to multiple reflection and interference effects. The structure can be fabricated by micro/nanomanufacturing by coating a wafer with a metallic gold film first and then fabricating the grating structures. Further details regarding the emitter’s design methodology are given in the Methods section. For the top grating, the slit is filled with air, while for the bottom grating, the slit is assumed to be filled with the same material as the dielectric, which could be an infrared transparent polymer. For example, polyethylene exhibits a large penetration depth in the mid-infrared region and can be considered transparent given the microscale thickness of the proposed structure [31]. The refractive index of the dielectric film is assumed to be *n* = 1.5. The effect of having a small loss in the dielectric film is discussed, as provided in Supplementary Materials. The twist angle *β* between the top and bottom gratings is adjustable depending on the desired polarization state of thermal emission.

While the wave diffraction phenomena in subwavelength gratings are complex [2], the gratings in the present study may be approximated as an anisotropic thin film that displays birefringence and dichroism effects. The structure exhibits polarization-dependent absorption/emission due to its anisotropic characteristics. Electromagnetic waves undergo multiple reflections between interfaces, leading to cumulative phase and amplitude shifts. The symmetry of the structure is broken by introducing a twist angle between the two gratings. This chiral arrangement results in elliptical absorption/emission patterns. By tailoring the structural parameters and taking advantage of the constructive/destructive interference effect, the design achieves two key goals: (1) a notable degree of polarized thermal emission, and (2) when the twist angle is adjusted, the conditions for constructive/destructive interference shift from one polarization to another. Remarkably, the high degree of polarization is retained even as the polarization state is altered. Noted that though a preliminary design was obtained by the anisotropic model, the final parameters are optimized and fine-tuned using FDTD employing a more confined search range.

For a completely polarized plain wave, the state of polarization can be characterized by the polarization ellipse using the rotation angle *ψ* (0 ≤ *ψ* < *π*) and the ellipticity *χ* (–*π*/4 ≤ *χ* < *π*/4) [32]. Alternatively, it can be represented by a point (2*ψ*, 2*χ*) on the surface of Poincaré sphere as shown in Figure 1b. Generally, thermal emission is partially polarized, consisting of a polarized and an unpolarized component, such that the polarization state will lie inside the Poincaré sphere. The Stokes parameters of thermal emission can be calculated in terms of the polarized emissivity *ϵ* as [33]

(1) $$\left[\begin{array}{c} S_{0}\\ S_{1}\\ S_{2}\\ S_{3} \end{array}\right]=\frac{S_{0,\text{bb}}}{2}\left[\begin{array}{c} \epsilon_{0,0}+\epsilon_{\pi /2,0}\\ \epsilon_{0,0}-\epsilon_{\pi /2,0}\\ \epsilon_{\pi /4,0}-\epsilon_{3\pi /4,0}\\ \epsilon_{0,\pi /4}-\epsilon_{\pi /2,-\pi /4} \end{array}\right]$$

where *S*_0,bb_ is the first Stokes parameter of blackbody emission at the emitter temperature and has been expressed in Ref. [11], and the subscripts of *ϵ* represent the parameters (*ψ*, *χ*). A total of six polarized emissivities are needed to determine the Stokes parameters. The polarized emissivity can be directly calculated using fluctuational electrodynamics or obtained indirectly from the polarized absorptivity (*i.e.*, one minus the reflectance), as described by Kirchhoff’s law [2].

Given an electromagnetic wave whose polarization state $\left|A^{+}\right\rangle$ is specified by its polarization parameters (*ψ*, *χ*), the orthogonal polarization state $\left|A^{-}\right\rangle$ can be expressed by polarization parameters (*ψ* + *π*/2, –*χ*). The orthogonality is denoted by $\left\langle A^{+}|A^{-}\right\rangle =0$ . Here, *ϵ*_+_ and *ϵ*_–_ are used to denote a pair of elliptically polarized emissivity with mutually orthogonal polarizations characterized by (*ψ*, *χ*) and (*ψ* + *π*/2, –*χ*), respectively. To demonstrate full-Stokes thermal emission over the Poincaré sphere, polarized emissivity of four polarization states (1–4) and their orthogonal polarization are chosen, located at various longitudes and latitudes on the spherical surface to examine the performance of the designed structure. State 1 (*ψ* = *π*/2, *χ* = 0) and state 2 (*ψ* = *π*/4, *χ* = 0) represent linear polarizations with different rotation angles. State 3 (*ψ* = *π*/2, *χ* = – *π*/8) represents elliptical polarization, while state 4 (*ψ* = *π*/2, *χ* = – *π*/4) represents circular polarization.

**Figure 2:**
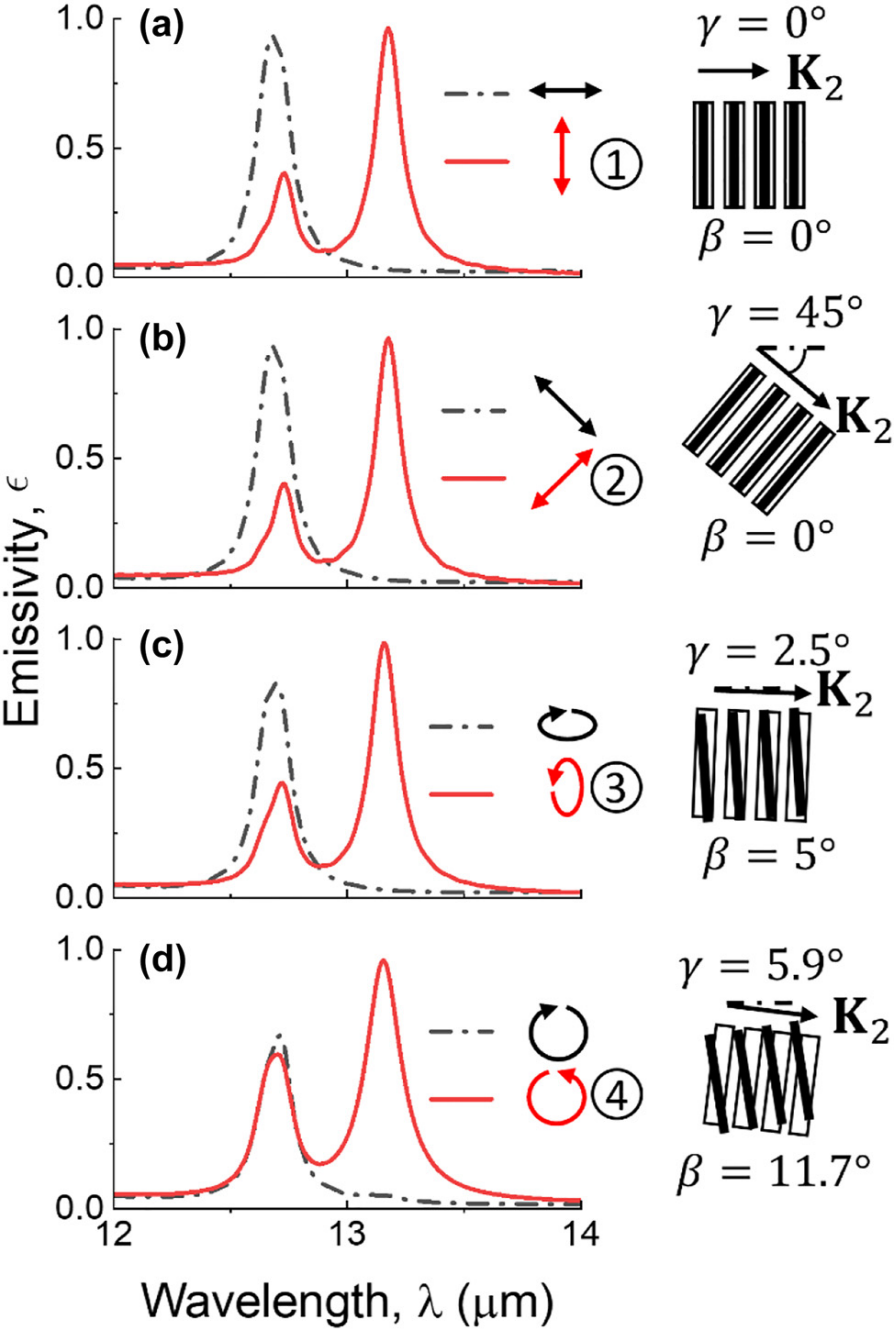
Spectra of polarized emissivity *ϵ* of the emitter with varying orientations of *x*-axis and twist angles *β*. The emissivity of the twisted-gratings structure is tuned for (a) state 1, (b) state 2, (c) state 3, and (d) state 4. The polarizations of the spectral emissivity are indicated by the arrows. The insets on the right illustrate the orientation of bottom grating vector **K**_2_ (the angle is denoted by *γ*) and specify twist angle of the gratings.

The spectra of polarized emissivity (in the normal direction) for the emitters designed for states 1 to 4 with its orthogonal polarized emissivity are plotted in Figure 2a–d, respectively. The emissivity is calculated indirectly from the absorptivity using lumerical FDTD (refer to the Methods section for the simulation setup). The colored arrows indicate the polarization of the electric field in the same coordinates as the polarization ellipse shown in Figure 1b. A schematic is depicted on the right side to illustrate the twist angle *β* and the orientation of bottom grating vector **K**_2_ in each case (the orientation angle is denoted by *γ*). Note that rotating **K**_2_ merely changes the rotation angle *ψ*, as seen from Figure 2a and b. For unaligned gratings, *ψ* depends on the orientation of **K**_2_ and the twist angle between the gratings. However, the ellipticity *χ* at a given wavelength depends on the twist angle *β* for specified grating parameters. Therefore, the tunability of the polarization state of thermal emission can be realized by adjusting **K**_2_ orientation and twist angle. Figure 2 shows that the emission peak remains at nearly the same wavelength, *λ* ≈ 13.17 µm for all four cases, with a dichroism between *ϵ*_+_ and *ϵ*_–_ exceeding 0.93. As *β* increases, the state of polarization is continuously modulated from linear to nearly circular, through intermediate elliptically polarized states. The maximum degree of polarized emission for state 3 and 4 is achieved at *β* = 5° and 11.7°, respectively. Negative twist angle *β* can be achieved with a structure that has the opposite chirality, thereby yielding an opposite *χ*. By altering the orientation and the twist angle *β*, the proposed emitter can realize high thermal emission dichroism of arbitrary polarization on the Poincaré sphere, thus enabling full-Stokes thermal emission with desired polarization states.

The polarization state of the emitter is analyzed in order to identify an optimal *β* for the desired *χ*. The degree of polarization (DoP), given by $\sqrt{S_{1}^{2}+S_{2}^{2}+S_{3}^{2}}/S_{0}$ , and the degree of circular polarization (DoCP), given by $\left|S_{3}\right|/S_{0}$ , are commonly used to quantify the polarized and circularly polarized portion, respectively. The concept of elliptical dichroism has been brought up by several research groups [34, 35] and finds frequent application in biomedical analysis to describe the absorption phenomena of elliptically polarized light. In this work, relative emission dichroism (RED) between two generally elliptically polarized emissivity *ϵ*_+_ and *ϵ*_–_ of arbitrary ellipticity *χ* is defined as follows:

(2) $$\text{RED}\left(\chi \right)=\frac{\left|\epsilon_{+}-\epsilon_{-}\right|}{\epsilon_{+}+\epsilon_{-}}$$

**Figure 3:**
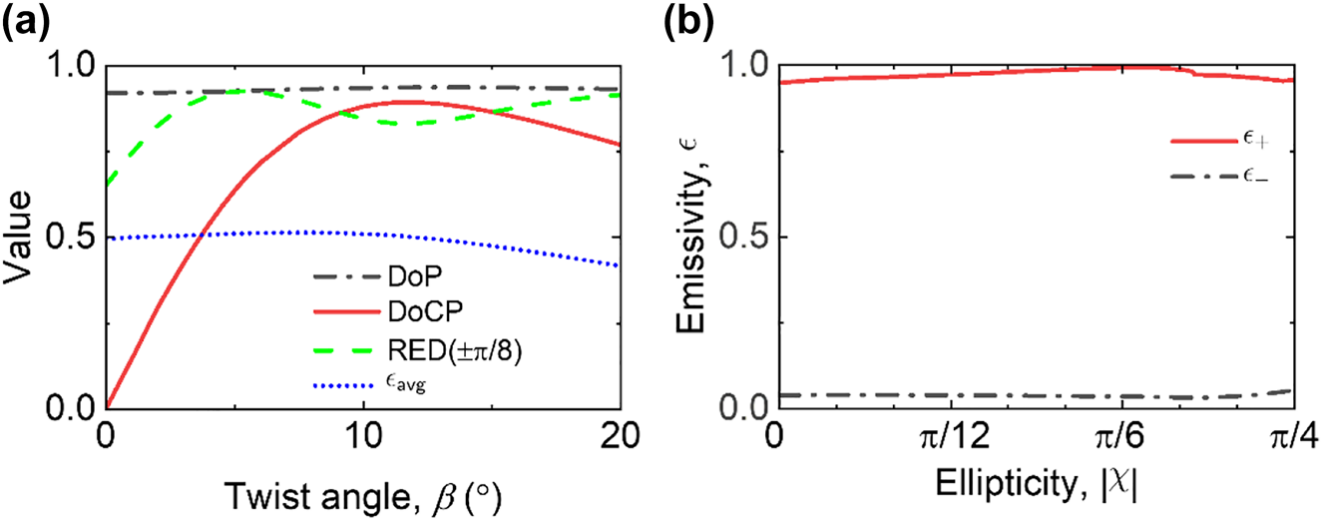
The polarization characteristic parameters of the emitter with varying twist angle *β*, and the spectral emissivity *ϵ* as the function of the desired ellipticity |*χ|* obtained by modifying the emitter twist angle. (a) The degree of polarization (DoP), degree of circular polarization (DoCP), relative emission dichroism (RED) at an ellipticity of |*χ*| = *π*/8, and the average emissivity *ϵ*_avg_ at *λ* = 13.17 µm as a function of *β*. (b) Spectral emissivity at *λ* = 13.17 µm as a function of |*χ*|, where the optimal *β* is chosen for each |*χ*|.

Here, RED is used to characterize the effectiveness in emitting the elliptically polarized thermal emission of arbitrary *χ*. In Eq. (2), *ψ* is chosen to maximize the emission dichroism (*ϵ*_+_ – *ϵ*_–_); thus, RED depends on *χ* only, and RED(–*χ*) = RED(*χ*). The value of RED lies between 0 and DoP. For instance, if *χ* = ±*π*/4 is chosen, RED(±*π*/4) would be the relative circular dichroism that equals DoCP. On the other hand, when *χ* is equal to the ellipticity ±χ* of the polarized portion of thermal emission (*i.e.*, $\sin2\chi^{*}=S_{3}/\sqrt{S_{1}^{2}+S_{2}^{2}+S_{3}^{2}}$ ), RED(±χ*) is exactly DoP. In Figure 3a, DoP, DoCP, RED(±*π*/8), and the average emissivity given by *ϵ*_avg_ = (*ϵ*_+_ + *ϵ*_–_)/2 at *λ* = 13.17 µm are plotted as a function of the twist angle. The DoP remains high for 0° < *β* < 20°, while DoCP varies. This indicates that the ratio of polarized emission to total emission remains nearly constant while the polarization state changes. The optimal *β* for linearly polarized emission (*χ* = 0) is obtained at *β* = 0° with a DoCP of zero. For left-handed circularly polarized emission (*χ* = – *π*/4), *β* = 11.7° is selected, resulting in the highest DoCP of 0.90. It should be noted that at *β* = 11.7°, DoP is slightly higher than DoCP, suggesting that the polarized portion of thermal emission is marginally elliptical. In addition, elliptical polarization is acquired for 0° < *β* < 11.7°, with each *χ* having an optimal *β*. Taking *χ* = ±*π*/8 as an example, two peaks can be observed in the RED curve at *β* = 5° and *β* = 20°. However, the smaller *β* with higher *ϵ*_avg_ is selected to be optimal since a higher emissivity is preferable as a light source. This approach can also be applied to determine the appropriate *β* for other *χ*. By carefully selecting the optimal *β*, polarized emissivity of orthogonal polarizations is obtained as a function of |*χ*|. Figure 3b plots *ϵ*_+_ and *ϵ*_–_ as a function of |*χ*| when the emission dichroism is optimized. An average RED of 0.92 across |*χ*| is achieved through the searching process. A slight decrease in emission dichroism occurs around *χ* = ±*π*/4 due to the imperfection of circular polarization at *β* = 11.7°. The details of the change of polarization and the trade-offs between different performance is provided in Supplementary Materials.

**Figure 4:**
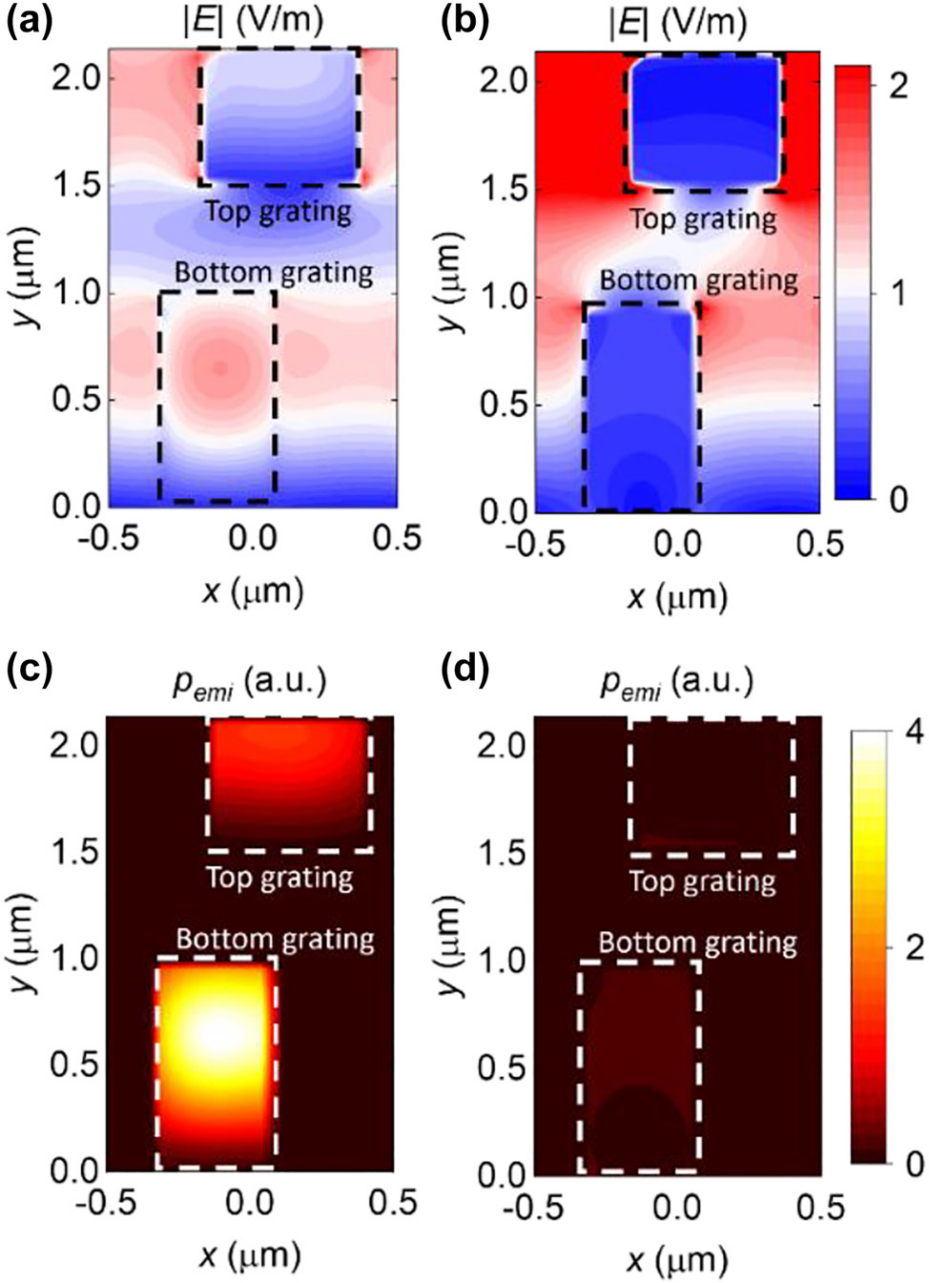
Electric field |*E*| and emission density *p*_emi_ in the *x–z* plane at *λ* = 13.17 µm. Electric field distribution for (a) LCP incidence and (b) RCP incidence. The emission density distribution for (c) LCP and (d) RCP emission. The emission density is obtained from the absorption density calculated from local energy dissipation.

According to the local Kirchhoff law [36], the emissivity density *p*_emi_ of a reciprocal medium is equal to the absorption cross-section density. The latter can be calculated from the local energy dissipation rate (or the absorption density) response to an incident electromagnetic wave. The absorption density is calculated from the local electric field by *p*_abs_ = *ε″ε*_0_ *ω*|*E*|^2^/2 [37], where *ε*_0_ is the permittivity of vacuum and *ε″* is the imaginary part of the dielectric function, and *ω* is the angular frequency. The details of the calculation can be found in Supplementary Materials. Figure 4a and b show the electric field distributions in the twisted-gratings structure designed for state 4 at *λ* = 13.17 µm for a left-handed circularly polarized (LCP) and right-handed circularly polarized (RCP) incidence with unity amplitude, respectively, at normal incidence. For LCP incidence, an intense electric field is centered at the entire bottom layer, while a relatively weaker field is centered at the top SiC strips. Conversely, although a comparably strong electric field can be evoked under RCP incidence, it is only concentrated in the dielectric or air, with nearly zero distributions inside the SiC strips. These distinct electromagnetic responses between LCP and RCP result in circular dichroism of the structure. The emission density is plotted in Figure 4c and d for LCP and RCP emission, respectively. The emission density is zero outside the SiC strips since air and the dielectric are lossless. Only LCP exhibits a high-density distribution, indicating that the SiC strips in both gratings contribute to the total polarized emission, although most of the thermal emission is generated by the bottom SiC strips.

**Figure 5:**
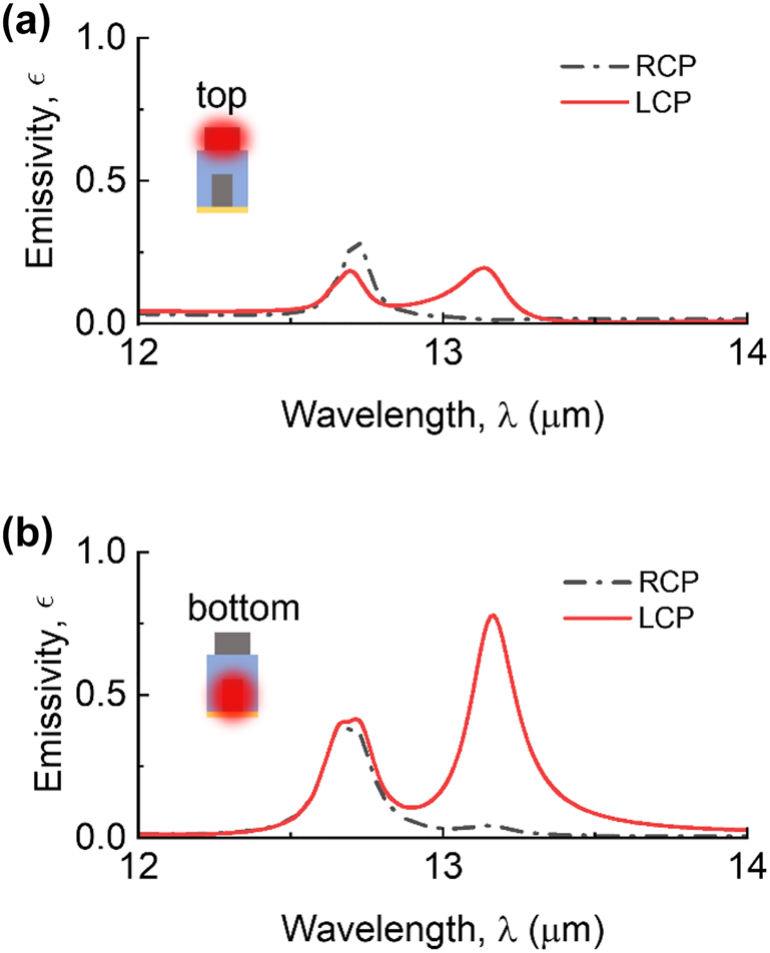
The spectra of polarized emissivity from the individually heated SiC gratings. The spectral polarized emissivity *ϵ* is contributed by (a) the top SiC grating, or (b) the bottom SiC grating, while the rest of the structure is maintained at absolute zero temperature.

Thermal emission form layered structures with a nonuniform temperature distribution can be evaluated by the superposition of the intensities emitted by each layer [38]. For plasmonic metasurfaces supporting electromagnetic resonances, e.g., surface plasmon polaritons, surface phonon polaritons, etc., the energy dissipation is usually concentrated on the surface or edge of the metallic meta-atoms [17, 39]. Such that the temperature nonuniformity may result in a degraded DoP of plasmonic-based polarized thermal emitters due to a relatively lower temperature on the surface of meta-atoms. Recent studies have shown the advantages of the localized Joule heating method for emissivity measurement [40, 41], including a fast modulation rate, robustness against background noises. Accordingly, one may be interested in the performance of the twisted-gratings structure with high temperature uniformity. Figure 5a and b reveal the spectral polarized emissivity when either the top or bottom SiC grating is heated, while the rest of the structure is maintained at absolute zero temperature. The derivation of the emissivity can be found in Supplementary Materials. A polarized thermal emission can be observed in both scenarios. At *λ* = 13.17 µm, the top layer emission scenario exhibits a DoCP of 0.88, while the bottom layer emission scenario exhibits a DoCP of 0.90. In other words, thermal emission from the proposed structure is insensitive to localized heating and maintains a high degree of polarization under a substantial level of temperature non-uniformity. From an emission perspective, unpolarized thermal emissions are generated throughout the SiC strips and undergo multiple reflections process between the boundaries. Waves of different polarizations are subject to interference effects; as a result, only a desired polarization can eventually leave the structure, while the orthogonal polarization is reabsorbed by the SiC strips.

**Figure 6:**
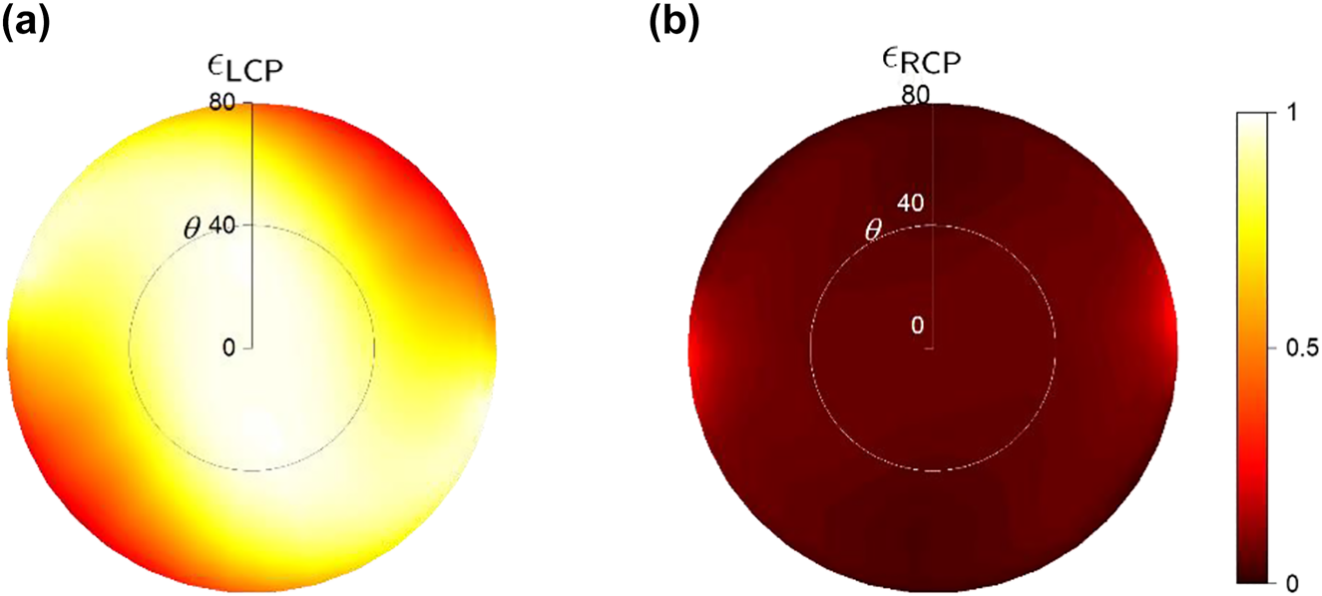
The effect of emission angle on the performance of a full-Stokes thermal emitter is examined. The emissivity for left-hand circularly polarized (LCP) emission is shown in (a), and the emissivity for right-handed circularly polarized (RCP) emission is shown in (b). These plots are presented as functions of the azimuthal angle *θ* and polar angle, based on the scenario depicted in Figure 2d.

The performance of the proposed emitter is not limited to normal emission. Taking the structure designed for state 4 as an example, the contours of angular emissivity for LCP and RCP at *λ* = 13.17 µm are plotted in Figure 6a and b, respectively. One can observe that thermal emission is highly polarized in the angular region of 0° < *θ* < 35° with an average DoCP of 0.87 at *θ* = 35°. Such omnidirectional performance can be applied in related applications that require a wide-angle illumination.

**Figure 7:**
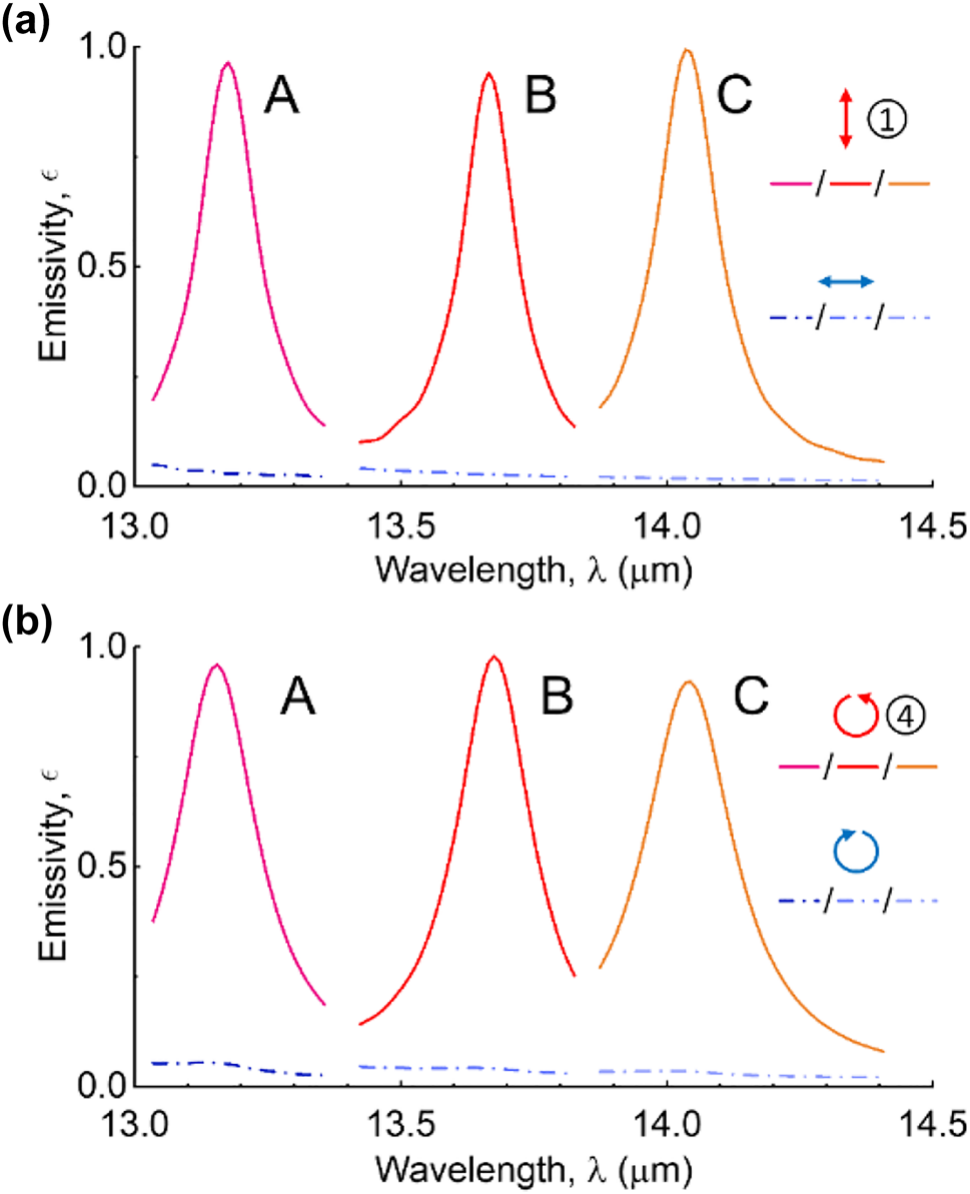
Polarized emissivity is analyzed for the full-Stokes emitters designed at various wavelengths. The spectral of the polarized emissivities designed for (a) state 1 with zero *β* for three emitters A, B, and C, and for (b) state 4 with *β*_B_ = 18.7° and *β*_C_ = 18.6° for emitters B and C, respectively. The original structure is denoted as A, while emitters B and C are designed at *λ* = 13.68 µm and *λ* = 14.05 µm, respectively.

The twist-gratings structure can be designed to operate at different wavelengths with the modification of the geometrical parameters. Two different structures denoted by B and C (A represents the original structure), are optimized to realize full-Stokes thermal emission with desired polarization states at wavelengths of approximately 13.68 µm and 14.05 µm, respectively, as illustrated by Figure 7. The geometrical parameters of the emitters are given as follows: for B: Λ_1_ = Λ_2_ = 1 µm, *w*_1_ = 0.39 µm, *t*_1_ = 1.0 µm, *d* = 2.4 µm, *w*_2_ = 0.49 µm, *t*_2_ = 2.5 µm, and for C: Λ_1_ = Λ_2_ = 1 µm, *w*_1_ = 0.53 µm, *t*_1_ = 1.0 µm, *d* = 1.9 µm, *w*_2_ = 0.47 µm, *t*_2_ = 3.0 µm. Figure 7a demonstrates the polarized emissivities designed for state 1 with zero *β* for the emitters, and Figure 7b discloses polarized emissivities designed for state 4 with *β*_B_ = 18.7° and *β*_C_ = 18.6° for emitters B and C, respectively. Similarly, elliptically polarized emission can be achieved by adjusting the value of *β*. In principle, the proposed design methodology allows for the realization of full-Stokes thermal emission with desired polarization states over a range of mid-infrared wavelengths.

## 3 Methods

### 3.1 Design and optimization

A grating structure with a deep subwavelength grating period could be considered as homogeneous and anisotropic with good precision, as per the effective medium theory (EMT) [2]. The absorption can be calculated using the transfer matrix method (TMM) [42]. However, concerning the feasibility of fabricating the gratings on a wafer scale and reducing the number of parameters to optimize in the simulation, this study employs a fixed grating period of 1 µm. Nevertheless, this choice of grating period could result in a minor deviation in EMT calculation from the rigorous method, particularly for transverse magnetic (TM) incidence, which excites surface resonance. An alternative solution is provided by Full-wave simulation, but the high computational complexity impedes the utilization of mass optimization algorithms. This study combines particle swarm optimization (PSO) (a method that iteratively optimizes the problem using repetitive computational generations) with EMT-TMM to obtain the initial geometrical parameters of the full-Stokes emitter. Subsequently, PSO is incorporated with full-wave simulation results (Lumerical FDTD) to fine-tune the geometrical parameters within a relatively narrow parameters range based on the preliminary result. The objective function of PSO is to maximize the mean value of RED across varying ellipticity. It is worth mentioning that the machine learning and deep learning could be used to perform the inverse design and optimization of nanostructured metamaterials [43, 44]. The computational cost could be significantly reduced with a well-trained surrogated model, offering advantages in terms of the tunability to a targeted performance.

### 3.2 Numerical simulations

Results of emissivity are obtained by absorptivity using lumerical FDTD (Lumerical Inc.). The wavelength-dependent dielectric function of SiC is expressed by a Lorentz model [45] and gold is described by Drude model [2]. One-period unit cell is used in simulation with a plane wave sources incidence. For a non-zero twist angle *β*, the whole structure is rotated about *z*-axis by an angle of –*β*/2 to equalize the lattice constants of the top and bottom gratings. For normal incidence, periodic in-plane boundary conditions and perfectly matched layer (PML) out-of-plane boundary conditions are used; since the reflected wave has near-zero off-normal components, the TE and TM waves are impinged separately, and the average electric fields are recorded to calculate the corresponding Fresnel coefficients in a specified elliptical basis. For oblique incidence, Bloch in-plane boundary conditions are used with a narrow-band source; two orthogonal polarized linear polarization plane waves with a relative phase retardance are combined as the elliptical polarized incidence, and absorptivity is directly obtained from Poynting vector.

## 4 Conclusions

This study demonstrates the use of a double-layer twisted-gratings structure to achieve full-Stokes thermal emission with desired polarization states in the mid-infrared region. The polarization state of thermal emission can be continuously tuned by adjusting the twist angle between the gratings and the orientation of the emitter. This enables the feasible polarization states to nearly cover the entire Poincaré sphere with an average relative emission dichroism of 0.92. Moreover, the proposed emitter can produce a high degree of circularly polarized emission even with a temperature difference in different layers. The twisted-grating structure can function over a wide range of emission angles (0° < *θ* < 35°). The structure’s geometry can be varied to allow functionality at different mid-infrared wavelengths. This study presents a promising design for realizing full-Stokes thermal emitters in a compact device with diverse applications such as drug analysis, mid-infrared imaging, and ellipsometry.

## Supplementary Material

Supplementary Material Details
