# Supplementary material for: Tailoring full-Stokes thermal emission from twisted-gratings structures
Source: Nanophotonics. 2023 Oct 25;13(5):803–11. doi: 10.1515/nanoph-2023-0395 (PMC11501260; doi:10.1515/nanoph-2023-0395)
Supplement: Supplementary file 1 — Supplementary Material Details [file j_nanoph-2023-0395_suppl_001.pdf]

Supplementary Materials:

Tailoring full-Stokes thermal emission from twisted-gratings structures

Chiyu Yang,[1] Wenshan Cai,[2] and Zhuomin M. Zhang[1,*]

[1] 1George W. Woodruff School of Mechanical Engineering Georgia Institute of Technology, Atlanta, GA 30332, USA
[2] School of Electrical and Computer Engineering, Georgia Institute of Technology, Atlanta, GA 30332, USA
[*] zhuomin.zhang@me.gatech.edu

1 The effect of small but non-zero imaginary part of the dielectric constant

Since a very small but non-zero imaginary part of the dielectric constant can lead to significant deviations in simulations, especially in devices with high Q-factors, a constant refractive index of $n = 1.5 + 0.01\text{i}$ for the dielectric layer is employed in the context of Figure 2d to examine the effect of loss on the emissivity. The comparison results are depicted in Figure S1. In the case of a dielectric possessing a non-zero imaginary part, specifically $n'' = 0.01$, the emissivity of LCP remains nearly unaffected at the designated wavelength. Conversely, the emissivity of RCP undergoes an increase from 0.05 to 0.08, indicating a decrease in the Degree of Circular Polarization from 0.90 to 0.85. It should be noted that real materials, such as polyethylene, typically have a much lower n'' than 0.01. Therefore, the assumption of loss dielectric layer is justified.

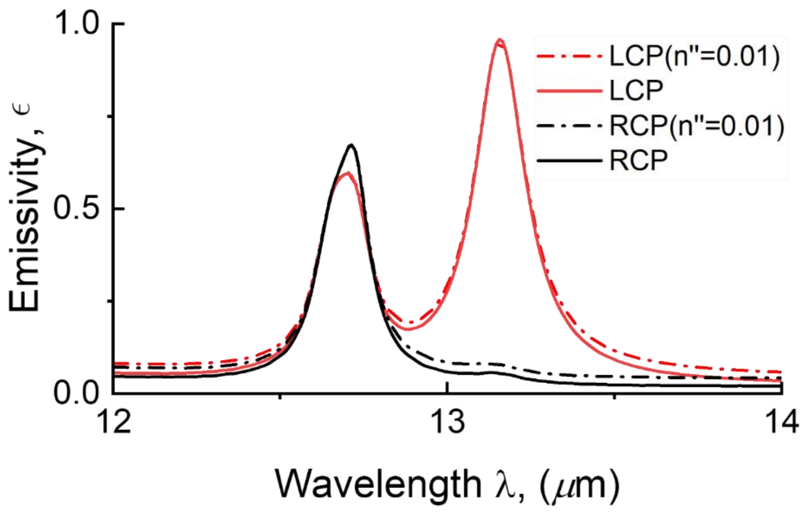

Figure S1. The emissivity of the structure with lossless dielectric material (solid line) and the emissivity of the structure with the dielectric material has a refractive index of $n = 1.5 + 0.01\text{i}$ employed for the scenario of Figure 2d (dash-dotted line).

2 Path of state of polarization on Poincaré sphere

The path of the state of polarization at λ_{design} = 13.17 μm, achieved by changing parameters (ψ, χ), is traced on Poincaré sphere in Figure S2. The locations as indicated by (a) to (d) correspond to scenarios for achieving states 1–4, respectively. The octant is nearly covered as 2ψ varies from 90° to 180°, as shown from the comparison between (a) and (b), and 2χ varies from 0° to –74.0°, as shown by comparing (a), (c), and (d). It is noteworthy that the geometry is designed to optimize the average relative emission dichroism (RED) for the desired emission state. A trade-off exists in the optimization process, balancing higher emission dichroism against a larger area to be covered on the Poincaré sphere.

Another example, characterized by parameters $\Lambda_1 = \Lambda_2$ = 1 μm, w_1 = 0.40 μm, t_1 = 0.60 μm, d = 0.59 μm, w_2 = 0.30 μm, t_2 = 0.96 μm, β = 0° to –11.7°, is designed to maximize the coverage area at λ_{design} = 13.05 μm, as demonstrated in Figure S3. The path of the state of polarization can cover a large solid angle on the Poincaré sphere (90° ≤ 2ψ ≤ 180° and –87.4° ≤ 2χ ≤ 0°), although the average RED is not maximized at this wavelength. In general, the proposed design can nearly cover the Poincaré sphere with a high degree of polarization.

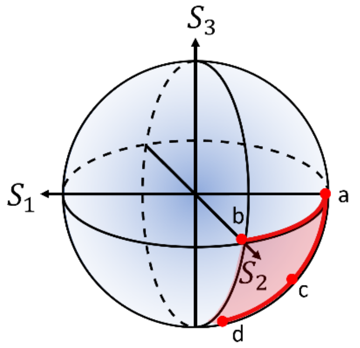

	β, γ	DoP	Ψ, X
a	0°, 0°	0.92	90°, 0°
b	0°, 45°	0.92	45°, 0°
c	5°, 2.5°	0.93	90°, −22.5°
d	11.7°, 5.9°	0.93	90°, −37.0°

Figure S2. The path of the state of polarization on the Poincaré sphere is plotted. The emitter is designed to maximize the average relative emission dichroism (RED) at λ_{design} = 13.17 μm. The locations (a) through (d) correspond to the scenarios for achieving states 1 to 4, respectively. The angle parameters of the emitter and the polarization parameters of the emission are provided on the right side of the figure.

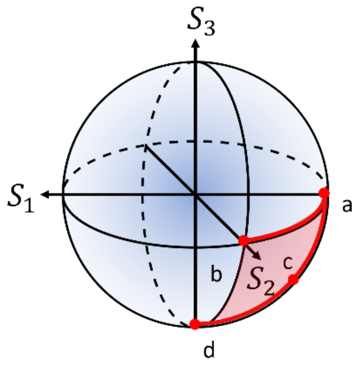

	β, γ	DoP	Ψ, X
a	0°, 0°	0.92	90°, 0°
b	0°, 45°	0.92	45°, 0°
c	5.5°, 2.8°	0.92	90°, −24.6°
d	11.7°, 5.9°	0.92	90°, −43.7°

Figure S3. The path of the state of polarization on the Poincaré sphere for the emitter designed to maximize the coverage area at λ_{design} = 13.05 μm. The geometrical parameters are $\Lambda_1 = \Lambda_2$ = 1 μm, w_1 = 0.40 μm, t_1 = 0.60 μm, d = 0.59 μm, w_2 = 0.30 μm, t_2 = 0.96 μm, β varies from 0° to 11.7°. The locations (a) through (d) correspond to the scenarios for achieving states 1 to 4, respectively. The angle parameters of the emitter and the polarization parameters of the emission are provided on the right side of the figure.

3 Emission density and emissivity of individually heated gratings

Consider a scenario where a plane wave $E_{inc}(\omega)$ of arbitrary polarization propagating in $\mathbf{k}_{\text{inc}}$ direction is impinged on a finite-size body with volume *V*, the total power absorbed by the body is given by the dissipation rate per unit volume (or the absorption density p_{abs}) as

$$\int_V p_{abs}(\mathbf{r},\omega)d^3\mathbf{r} = \int_V \text{Im}[\varepsilon(\mathbf{r},\omega)]\frac{\omega\varepsilon_0}{2}\left|\mathbf{E}(\mathbf{r},\omega)\right|^2 d^3\mathbf{r} \tag{S1}$$

where $\varepsilon(\mathbf{r},\omega)$ is the relative permittivity and $\mathbf{E}(\mathbf{r},\omega)$ is the field in the structure illuminated by $E_{inc}(\omega)$. A general approach to characterize the local absorption of a plane wave by a finite-size body is to use the absorption cross section σ_{abs}, or the absorption cross section density $\alpha_{abs}(\mathbf{r})$ given by $\sigma_{abs} = \int_V \alpha_{abs}(\mathbf{r})d^3\mathbf{r}$. One can relate the absorption cross-section density α_{abs} with the absorption density p_{abs} as

$$\alpha_{abs}(\mathbf{k}_{inc,}\mathbf{r},\omega)\frac{c\varepsilon_0}{2}\left|E_{inc}(\omega)\right|^2 = p_{abs}(\mathbf{r},\omega) \tag{S2}$$

where $\frac{c\varepsilon_0}{2}\left|E_{inc}(\omega)\right|^2$ is magnitude of the Poynting vector of the incident wave. The local Kirchhoff law states that for a reciprocal medium, the quantities of absorption cross-section density α_{abs} in $\mathbf{k}_{\text{inc}}$ direction and the emissivity density η in $-\mathbf{k}_{\text{inc}}$ direction are equal [1], that is,

$$\alpha_{abs}(\mathbf{k}_{inc,}\mathbf{r},\omega) = \eta(-\mathbf{k}_{inc,}\mathbf{r},\omega) \tag{S3}$$

For a heated body with a temperature distribution $T(\mathbf{r})$, the power emitted by the body in solid angle $d\Omega$ can be cast in the form of

$$P_{emi}(\omega) = \int_V \eta(-\mathbf{k}_{inc,}\mathbf{r},\omega)\frac{I_b[\omega,T(\mathbf{r})]}{2}d^3\mathbf{r}d\Omega \tag{S4}$$

where $I_b[\omega,T(\mathbf{r})] = [\omega^2/(4\pi^3c^2)]\{\hbar\omega/[\exp(\hbar\omega/k_BT)-1]\}$ is the blackbody radiance. Based on Eqs. (S1) – (S4), the corresponding emission density (power emitted per unit volume in the $-\mathbf{k}_{\text{inc}}$ direction with a solid angle $d\Omega$) becomes

$$p_{emi}(\mathbf{r},\omega) = \{p_{abs}(\mathbf{r},\omega)I_b[\omega,T(\mathbf{r})]\}\frac{1}{c\varepsilon_0\left|E_{inc}(\omega)\right|^2}d\Omega \tag{S5}$$

The emissivity of individually heated grating is expressed as

$$\epsilon = \frac{\int_V p_{emi}(\mathbf{r},\omega)}{\int_V p_{emi,blackbody}(\mathbf{r},\omega)} \tag{S6}$$

For emissivity if one of the two gratings is heated up while the rest part remains at absolute zero temperature, Eq. (S6) can be written in a simple expression:

$$\epsilon = \int_{V'} p_{abs}(\mathbf{r},\omega) \tag{S7}$$

where *V'* denotes the heated region.

1. J.-J. Greffet, P. Bouchon, G. Brucoli, and F. Marquier, "Light Emission by Nonequilibrium Bodies: Local Kirchhoff Law," *Phys. Rev. X*, vol. 8, no. 2, p. 021008, 2018.
